# E3 Ubiquitin Ligase TRIM Proteins, Cell Cycle and Mitosis

**DOI:** 10.3390/cells8050510

**Published:** 2019-05-27

**Authors:** Santina Venuto, Giuseppe Merla

**Affiliations:** 1 Division of Medical Genetics, Fondazione IRCCS Casa Sollievo della Sofferenza, Viale Padre Pio, 71013 San Giovanni Rotondo, Foggia, Italy; venutosantina@gmail.com; 2 PhD Program, Experimental and Regenerative Medicine, University of Foggia, Via A. Gramsci, 89/91, 71122 Foggia, Italy

**Keywords:** TRIMs, cell cycle, cancer, mitosis

## Abstract

The cell cycle is a series of events by which cellular components are accurately segregated into daughter cells, principally controlled by the oscillating activities of cyclin-dependent kinases (CDKs) and their co-activators. In eukaryotes, DNA replication is confined to a discrete synthesis phase while chromosome segregation occurs during mitosis. During mitosis, the chromosomes are pulled into each of the two daughter cells by the coordination of spindle microtubules, kinetochores, centromeres, and chromatin. These four functional units tie chromosomes to the microtubules, send signals to the cells when the attachment is completed and the division can proceed, and withstand the force generated by pulling the chromosomes to either daughter cell. Protein ubiquitination is a post-translational modification that plays a central role in cellular homeostasis. E3 ubiquitin ligases mediate the transfer of ubiquitin to substrate proteins determining their fate. One of the largest subfamilies of E3 ubiquitin ligases is the family of the tripartite motif (TRIM) proteins, whose dysregulation is associated with a variety of cellular processes and directly involved in human diseases and cancer. In this review we summarize the current knowledge and emerging concepts about TRIMs and their contribution to the correct regulation of cell cycle, describing how TRIMs control the cell cycle transition phases and their involvement in the different functional units of the mitotic process, along with implications in cancer progression.

## 1. Introduction

### Cell Cycle and Mitosis

**Figure 1:**
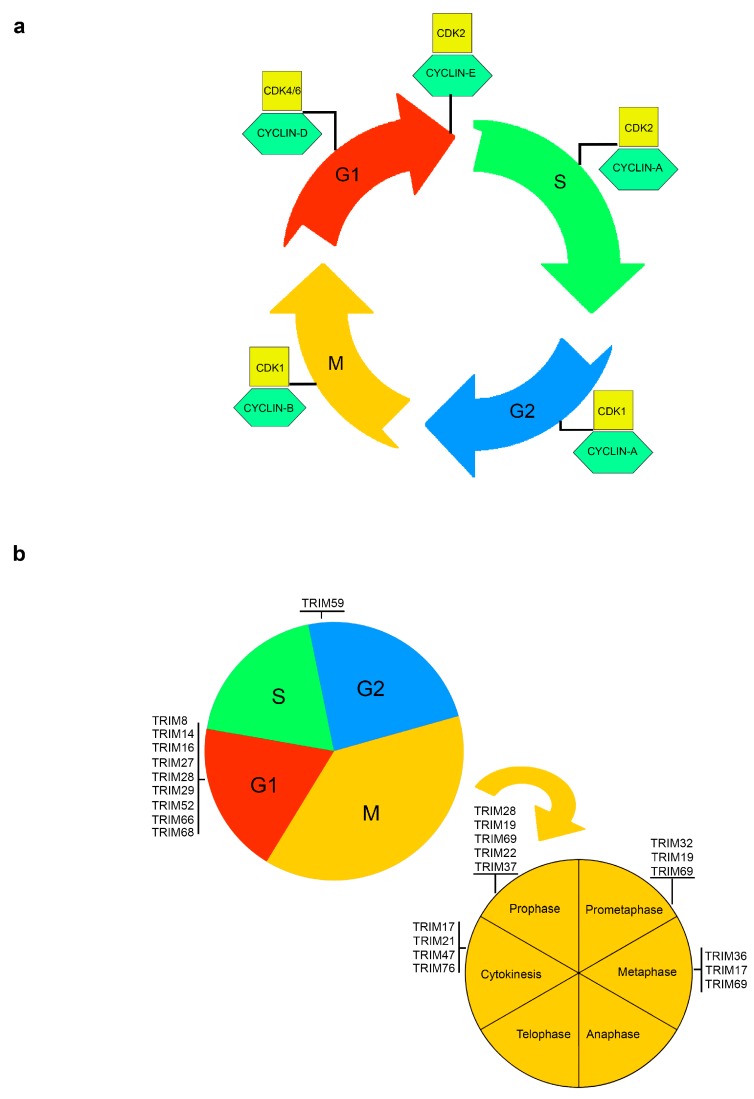
Tripartite motifs (TRIMs) regulate specific stages of cell cycle and mitosis. (**a**) Schematic representation of the cell cycle. Each of the main phases of the cell cycle—G1, S (when DNA synthesis occurs), G2 and mitosis—is controlled by CDKs, together with their regulatory partner proteins, the cyclins. Different phases of the cell cycle require different cyclins and the coordination between CDKs levels and the respective checkpoints prevent the entry into the following phase until cellular or genetic defects are repaired. (**b**) The critical and major TRIMs responsible of cell cycle phase transitions (up) and mitotic progression (down) are schematically represented within the phases they specifically are involved in.

Precise replication of genetic material and its equal distribution into daughter cells are essential to maintain genome stability. The eukaryotic cell cycle refers to the series of events comprising the sequential actions, during proliferation, of DNA synthesis (S-phase), and cell division (M-phase) with intervening gap phases to allow cell growth (G1-phase) and to check the integrity of genomic material (G2-phase). The normal cell cycle is driven by the coordinated and sequential rise and fall of CDKs activity and their regulatory partners, the cyclins. Different phases of the cell cycle require different cyclins and the transition through the cell cycle phases is governed by the respective checkpoints that prevent the entry into the next phase until cellular or genetic defects are repaired [1,2] (Figure 1a).

Among the different phases of the cell cycle, mitosis is a delicate event that must be executed with high fidelity to ensure genomic stability, since genetic material has to be duplicated and each chromosome must be segregated into two daughter cells. Each of the daughter cells must receive an exact copy of the genetic material, and defects in chromosome segregation has been linked to tumorigenesis [3]. 

The onset of mitosis is typically marked by nuclear envelope breakdown, condensation of the replicated DNA in chromosomes, and subsequently centrosomes separation, during prophase. Then, an increase in the frequency of microtubule shrinkage events allows the interaction between dynamic microtubule plus-ends and the condensed chromosomes. 

During prometaphase, the individualized chromosomes attach their kinetochores to the microtubules and congress to the center of the microtubule array. In addition to the kinetochore fibers, both the interpolar and the astral microtubules contribute to the spindle bipolar structure. When all of the chromosomes are bi-oriented and aligned, the cell is in metaphase, with sister kinetochores attached to microtubules from opposite spindle poles (bipolar attachment). The correct attachment is then stabilized, raising the alignment of sister chromatids at the metaphase plate, and followed by their segregation toward the opposite spindle poles in anaphase [4]. After successful chromosome segregation, the spindle microtubules undergo a dramatic reorganization, forming the spindle midzone. Telophase marks the reformation of the nuclear envelopes around daughter cells nuclei, as the cytokinetic furrow pinches the cell into two. Cytokinesis begins with the formation of the midbody, composed of the remnants of the spindle midzone and in the final step the plasma membranes resolve in a process called abscission [5]. In mammalian cells abscission fails if chromosomes are pulled apart erroneously or if the anaphase spindle midzone is not properly formed, leading to regression of the cleavage furrow and the formation of multinucleated cells [6,7,8]. 

## 2. TRIMs and Cell Cycle Progression

The tripartite motif (TRIM) family proteins, also known as RING, B-box, and coiled-coil (RBCC) family, are characterized by an N-terminal TRIM region containing three zinc-binding domains, a RING (R) domain and one or two B boxes (B1 box and B2 box), and a coiled-coil region [9]. There are more than 80 known TRIM protein genes in humans [10] that are involved in various cellular processes such as apoptosis, cell cycle regulation, viral response, cell proliferation, oncogenesis, and antiviral defense. Consistently, their alteration results in many distinct pathological conditions and a wide variety of diseases including cardiovascular, neurological, immunological diseases, musculoskeletal diseases, developmental disorders, as well as various forms of cancer [10,11,12,13,14,15,16].

The RING domain gives to the TRIM proteins a role as E3 ubiquitin ligases in the ubiquitination process [9,12]. In this system, TRIM proteins bind to the substrates determining their fate. The most common consequence is the ubiquitin-mediated protein degradation through the 26S proteasome, where the target proteins are ubiquitin-tagged and thus destroyed [17]. Together with proteasomal degradation, conjugation of ubiquitin to specific substrate proteins also modulates and ensures fidelity to many cellular processes, including intracellular signaling, innate immunity, transcription, autophagy, and carcinogenesis [10,12]. These different fates of the substrate proteins depend on the lysine of the ubiquitin moiety used for isopeptide bond formation [18,19,20,21,22]. For instance, K48-polyubiquitin chains manage proteasomal degradation, while K63-polyubiquitin conjugates are involved in non-proteasomal pathways, as intracellular signaling, DNA repair, and the endosomal–lysosomal system [23]. Of relevance, the control of the cell cycle, and in particular of mitosis, is one of the cellular processes that have to be faithfully coordinated and regulated by ubiquitination [5]. 

### 2.1. TRIMs Regulate Phase Transitions during the Cell Cycle

A number of TRIM proteins are involved in the control of cell growth and cell cycle transition phases (Figure 1b). A common effect of most TRIM proteins, when silenced or knocked-down, is the increase of the percentage of cells in G0/G1 and the reduction of the fraction of cells in S or G2-M phases. Specifically, TRIM8, TRIM14, TRIM27, TRIM28, TRIM29, TRIM52, TRIM59, TRIM66, and TRIM68 led to cell cycle arrest when depleted or silenced [24,25,26,27,28,29,30,31,32,33,34,35]. 

For an optimal cell cycle progression and cell proliferation, TRIM proteins act on crucial factors like p53 and/or on important pathways, including JAK/STAT signaling. The Janus kinase (JAK)/signal transducer and activator of transcription (STAT) pathway is a highly conserved cellular cascade which connects the extracellular signaling from growth factors and cytokines with the nuclear transcriptional machinery [36]. Cytokine-induced activation of the JAK/STAT3 pathway leads to JAK2 recruitment and STAT3 phosphorylation and dimerization. Then, the translocation of the STAT3 dimer from the cytoplasm to the nucleus alters the transcription of many genes that play a central role in proliferation, migration, survival, and resistance to apoptosis [37]. Deregulation of the JAK/STAT pathway leads to the activation of several genes involved in cell cycle progression, including cyclinB [38], p16, p21, p27, and others that control CDKs expression [39].

TRIM52 ablation, for instance, increases the level of activated p53 (pSer15) and one of its major targets, the cyclin-dependent kinase inhibitor p21. Induction of p21 by activated p53 inhibits cyclinE/CDK2 thereby preventing G1/S transition [40]. Additionally, TRIM28 knockdown significantly raises the expression of p21 and slightly alters p53 [24,26]. In human osteosarcoma cell lines, TRIM8 physically interacts, stabilizes, and activates p53 protein, resulting in a suppression of cell proliferation due to a p53-dependent cell cycle arrest in G1 [33]. Furthermore, both TRIM8 and TRIM29 may exert effect on cell cycle progression through their association with the JAK/STAT signaling pathway. TRIM29 knockdown increases the level of JAK2 and STAT3 phosphorylation, while TRIM8 interacts with and promotes STAT3 transcriptional activity with a consequent deregulation of STAT3 target genes that are directly involved in cell cycle progression [25,32]. Yet it was demonstrated that an enforced expression of TRIM14 leads to a decreased percentage of cells in the G1 phase along with an increase in S-phase cells [41]. 

### 2.2. TRIMs, Cell Cycle Transitions, and Cancer Progression 

#### 2.2.1. TRIMs Involved in Cell Cycle Control Regulate Cancer Progression

Many TRIMs regulating the cell cycle are involved in different types of cancer (Table 1). TRIM8 is downregulated in a number of tumors, including clear cells renal cell carcinoma (ccRCC), anaplastic thyroid carcinoma (ATC), and glioma (GM) [34,42]. In glioma tissues and cell lines, TRIM8 expression inversely correlates with tumor grade. Moreover, restored TRIM8 expression in patients glioma cell lines suppresses the tumor growth and induces a significant reduction of the clonogenic potential [43]. TRIM28 expression is positively correlated with cancer prognosis in specific cancer types as widely described in [44]. Higher TRIM28 gene expression has been linked to breast cancer, hepatocellular carcinoma (HCC), and prostate tumors [44,45,46]. 

Also, over-expression of TRIM28 is associated with poor outcome in GM patients [24], while TRIM52 controls cell cycle with a reduced proliferation speed and a compromised cycle progression in U87MG and A172 glioblastoma (GBM) cell lines [26]. Additionally, TRIM52 up-regulation promotes HCC cell proliferation, migration and invasion, is highly expressed in lung and colorectal cancer (CRC) cells and plays and oncogenic role in ovarian cancer (OC) [27,35,47,48]. TRIM68, TRIM27, and TRIM29 participate in the regulation of CRC tumorigenesis. TRIM29 negatively regulates migration and invasion of squamous cell carcinomas (SCC) and exerts an opposite oncogenic role in CRC, non-small cell lung carcinoma (NSCLC), lung squamous cancer and gastric cancer (GC) [25,49,50,51,52].

TRIM68 inhibits, while TRIM27 promotes CRC cells proliferation [53,54]. Additionally, TRIM27 is also involved in other types of cancer like intraductal carcinomas (IDCs) of salivary glands and ovarian cancers [55,56,57]. TRIM14 plays an oncogenic role and may be a prognostic factor and potential therapeutic target in osteosarcoma (OS). Upregulation of TRIM14 induces the growth, migration, and invasion of OS cells in vitro, promoting tumorigenesis [41]. TRIM14 is also up-regulated in human GBM and correlates with GBM progression with a specific role in tumor invasion and epithelial–mesenchymal transition (EMT) [58]. It is also correlated to CRC and GCs, promoting cell migration and invasion [59,60]. Yet, TRIM66 is overexpressed in NSCLC and controls invasion, migration, and proliferative capacities of lung cancer (LC) cells [61,62]. Then, in OS carcinogenesis, TRIM66 suppresses apoptosis pathway and promotes TGF-β signaling [30].

TRIM16 is central for the differentiation of neuroblastoma (NB) cells. Its overexpression decreases proliferation in NB, lung, breast, and skin cancer cell lines through a down regulation of cell cycle factors E2F1 and pRB [63]. Moreover TRIM16 has a tumor suppressor role, inhibiting the migration, invasion, and the EMT in OC cells and in HCC [64,65]. Finally, among TRIMs involved in cell cycle progression, TRIM59 plays and oncogenic role in a multiplicity of different cancers. TRIM59 protein levels are significantly high in non-small cell lung carcinoma (NSCLC) [29] and its expression is significantly elevated in cervical cancers, epithelial ovarian cancer (EOC), cholangiocarcinoma (CCA) tissues and cells, breast cancer, and CRC [28,66,67,68]. TRIM59 also serves as a pro-oncogenic protein in promoting the progression of renal cell carcinoma (RCC) [69].

#### 2.2.2. TRIMs Control Cancer Progression Regardless of Cell Cycle Regulation

Other TRIMs, although do not exert a specific role in the regulation of cell cycle phase transitions, are yet involved in controlling carcinogenesis and cancer progression (Table 1). Most of these TRIMs, like TRIM11 [70,71,72,73,74], TRIM23 [75], TRIM24 [76], TRIM25 [77,78,79], TRIM31 [80], TRIM37 [81], TRIM47 [82,83], TRIM65 [84], and TRIM71 [85] are up-regulated in cancer cells and tissues, playing an oncogenic role in different types of cancer. These TRIMs are often associated with unfavorable prognosis and promote cancer cells proliferation and migration, being often associated with proteins of central signaling pathways. On the other side, TRIMs like TRIM2 [86], TRIM3 [87,88], TRIM15 [89,90], TRIM26 [91], TRIM36 [92] TRIM45 [93], TRIM50 [94], TRIM58 [95,96], and TRIM62 [97] exert a tumor suppressor role, inhibiting cell proliferation and migration as well as promoting apoptosis. Finally, other TRIMs like TRIM32 [98,99,100] and TRIM44 play both an oncogenic and tumor suppressor role in different tumors [101,102,103,104,105,106,107].

The role of many TRIMs in cancer is closely related to their E3 ubiquitin ligase activity [78,80,82,93]. Among them, TRIM25 has an oncogenic role in colorectal cancer (CRC), gastric cancer (GC) and prostate cancer (PC) [77,78,79]. In PC TRIM25 is the E3 ubiquitin ligase of Ets related gene (ERG), an important transcription factor central for prostate cancers progression. TRIM25 as an ubiquitin ligase, targets ERG, mediating ERG polyubiquitination and stabilization in prostate cancer [78]. Then, TRIM31 is upregulated in HCC cells and exerts its oncogenic effect promoting the E3 ligase-mediated K48-linked ubiquitination and degradation of tuberous sclerosis complex (TSC) 1 and TSC2, the upstream suppressor of mTORC1 pathway. TRIM31 further overactivates mTORC1 oncogenic pathway [80]. Also TRIM45 exerts its tumor suppressor role using its E3 ligase activity to stabilize and activate p53 in glioma. In particular, TRIM45 conjugates K63-linked polyubiquitin chain to p53, thereby inhibiting the availability of p53 to the K48-linked polyubiquitination and consequent degradation [93]. Finally, TRIM47 plays an oncogenic role in CRC and PC [82,83]. It was recently reported that, as an E3 ubiquitin ligase, TRIM47 increases SMAD4 ubiquitination and consequent degradation. SMAD4 normally causes growth and invasion in human CRC cells, thus TRIM47 action inhibits CRC proliferation and metastasis [82].

## 3. TRIMs and the Mitotic Spindle Machinery

### 3.1. TRIMs and Their Relationship with the Centrosomes and Spindle Poles

In multicellular eukaryotes, the mitotic spindle is made up of microtubules extending from two opposing spindle poles. To ensure the accurate distribution of the DNA during mitosis, a specialized macromolecular complex termed the kinetochore assembles onto the centromere of each chromosome [114]. The kinetochore–microtubule interface is critically important, as depolymerization of kinetochore-associated microtubules ultimately provides the driving force for chromosome segregation [115]. The primary microtubules organizing centers in human cells are named centrosomes and are important for the regulation of normal cell cycle progression and cell division. During mitosis, centrosome duplication and subsequent separation guarantee the formation of the mitotic spindle and the proper chromosomal segregation, in order to form stabilized daughter cells with the correct amount of DNA in each cell [116]. The centrosome is duplicated once during a normal cell cycle, starting at the G1 phase and completed by the end of the G2 phase [117]. Deregulation of this duplication machinery increases the number of centrosomes, raising the formation of multipolar spindles and thereby leading to aneuploidy and chromosomal instability. Centrosomal amplification has been associated with the initiation and progression of multiple malignancies and is related to a more aggressive tumor biology [116]. Together with centrosomes, other organelles that ensure reliable segregation of chromosomes during mitosis are the spindle poles [118]. 

Notably, a subgroup of TRIM/RBCC proteins has been shown to share a common domain (COS/FN3/B30.2) at the C-terminal of the proteins, necessary for the microtubule binding [119]. TRIMs proteins RING-finger, B-Box, and coiled-coil (RBCC) domain can be found in isolation or in combination with C-terminal domains, including COS, FN3 and B30.2 domains. The COS domain is a two α-helical coils motif, responsible for dimerization and microtubule association [119]. The FN3 domain is a fibronectin type III motif that can contain DNA binding sites [120]. Finally, the B30.2 domain consists of a SPRY domain preceded by a region containing a “PRY” motif and is involved in protein-protein interactions [121].

TRIM proteins can control cell division during the transitions between the different phases of the cell cycle and a number of TRIM proteins co-localize with or are important for the regulation of key components of the mitotic spindle, including kinetochore, centrosomes and midbodies (Figure 1b).

Remarkably, some TRIM proteins localize at centrosomes and spindle poles and are important in the coordination of centrosome duplication and in the maintenance of the genome stability, ensuring a reliable chromosomes segregation [100,122,123,124,125,126]. Among them, TRIM28 and PML3/TRIM19 are associated with centrosomes and/or spindle poles and maintain genome integrity by preventing centrosome amplification through the control of the appropriate number of centrosome in mammalian cells [122,125]. Other TRIM proteins like TRIM69A and TRIM22 co-localize with centrosomes throughout the cell cycle and are essential for centrosome clustering [123,124], while TRIM32 localizes at spindle poles during mitosis [100]. Among TRIMs that have targets directly involved in the regulation of centrosomes clustering and spindle poles functions, TRIM28, TRIM19, and TRIM32 regulate NPM1, Aurora A, and MYCN, respectively. TRIM28 acts as an E3 SUMO ligase enhancing the SUMOylation and increasing the centrosome localization of NPM1, a nucleus and cytoplasm shuttle protein that coordinates centrosome duplication and whose SUMOylation contributes to centrosomal localization [125,127]. PML3/TRIM19 physically interacts with and represses Aurora A, a serine/threonine protein kinase whose overexpression induces centrosome amplification [128]. TRIM19 also colocalizes with α-tubulin at the spindle poles and its depletion leads to aberrant mitosis and genome instability [122]. 

TRIM32 localizes at the spindle poles during mitosis through a recruitment mediated by Cdk1/cyclin B phosphorylation [100]. In particular, TRIM32 and MYCN both localize at spindle poles where they physically interact, and TRIM32 rapidly poly-ubiquitinylates MYCN protein destining it to degradation via the proteasome system. Acting against MYCN, TRIM32 is a positive regulator of an important physiological process named asymmetric cell division (ACD), whose regulation is mediated by the spindle pole-associated ubiquitin-proteasome system [100,129]. Since misregulation of the ACD may lead to tumorigenesis [130], TRIM32 is also important for the induction of complete ACD, particularly in human neuroblastoma [100].

Other TRIMs control the centrosomes number with an activity directly related to their RING domain [124]. TRIM69A is associated with spindle poles and promotes centrosomal clustering, being essential for the formation and regulation of the mitotic spindle. TRIM69A plays a critical role in supporting mitotic fidelity, as TRIM69-depleted cells show a number of mitotic defects, including micronucleation and multipolar spindle formation, particularly in NSCLC [124]. As an E3-ligase, TRIM69A localization at centrosomes is dependent upon its E3-ligase activity through an intact RING domain [124]. Finally, TRIM37 plays an intriguing role in the regulation of centrosomal components, deregulating p53 stabilization and allowing cells to escape arrest following centrosome loss [126].

Overall these experimental data indicate that the co-localization of TRIMs with centrosome and spindle poles, together with their E3-ligase activity, regulate central component of the mitotic spindle machinery, via different mechanisms.

### 3.2. TRIMs Are Involved at the Kinetochore Level

The centromere is the site for the recruitment of the kinetochore, a large protein complex central for the alignment and segregation of chromosomes from the onset of mitosis, ensuring exclusive connection of sister chromatids to the opposite spindle poles. The kinetochore contains a large number of protein components that prevent erroneous attachment of microtubules, ensuring timely segregation during mitosis [131]. 

Among TRIM proteins, TRIM69, TRIM36, and TRIM17 play a role in chromosome segregation and cell-cycle regulation interacting with and regulating proteolysis and proteasome-dependent degradation of several kinetochore proteins [17,124,132]. 

TRIM69 is essential for the proper attachment of microtubules to kinetochores as its depletion increases BUBR1 occupancy at kinetochores, a sentinel protein component of the spindle assembly checkpoint (SAC) [124]. In the absence of proper microtubule-kinetochore attachments, the SAC inhibits the activity of the anaphase promoting complex/cyclosome (APC/C) [133].

TRIM36 and TRIM17 control kinetochore proteins proteolysis in different manners. TRIM36 specifically associates with the microtubule structure as it co-localizes with α-tubulin, one of the microtubule proteins, and has been identified as a novel specific centromere protein-H (CENP-H) binding protein [132]. TRIM36 not directly ubiquitinates and degrades CENP-H but may be involved in the proteolysis of CENP-H-associated proteins [132]. CENP-H is a centromere protein that has a central role in directing the assembly of the outer kinetochore and regulates the dynamics of microtubules that are embedded in kinetochores [134]. CENP-H aberrant expression causes chromosome mis-aggregation and plays an important role in the chromosomal instability frequently observed in cancers [135,136]. TRIM36 overexpression decelerates the cell cycle and attenuates cell growth, preventing the recruitment of CENP-H to centromeres. This evidence relates TRIM36-mediated control of kinetochore proteins with its effects on chromosomal instability and carcinogenesis [132]. 

TRIM17/(terf) interacts with the kinetochore protein ZWINT and degrades ZWINT protein in a proteasome dependent manner, through its ubiquitination [17]. Interestingly, it is known that ZWINT knockdown causes a chromosome bridge phenotype, abrogates the mitotic arrest and triggers cell death [137]. ZWINT also interacts with ZW10, a protein that is required for chromosome motility and spindle checkpoint control [138]. 

### 3.3. TRIMs at the End of Mitosis: A Possible Role at Midbody Level

TRIM proteins can also contribute to midbodies abundance, possibly through the autophagic removal of midbodies, immediately after cytokinesis. Midbodies are dense protein structures, containing the remnants of the cell division machinery [139]. They are formed at the site of abscission, are inherited by one of the two daughter cells and are removed by autophagy [139]. Several TRIMs like TRIM17, TRIM21, TRIM47, and TRIM76 control the number of midbodies per cell [140]. 

As an example TRIM17 co-localizes with or forms rings around midbodies [140]. TRIM17 knockdown causes an increase of midbodies number and size, indicative of less midbodies degradation that contributes to the autophagy-dependent removal of midbodies [140]. 

## 4. Conclusions

As discussed in this review, TRIM proteins regulate cell cycle progression and mitosis at different stages. Deregulation of TRIMs expression during cell cycle is directly related to an increase in the percentage of cells into a specific cell cycle phase (G1–S–G2 or M), modulating cell proliferation signals and resistance to cell death. Moreover, some TRIM proteins expression and localization is dependent on the progression and alternation of the different cell cycle phases. The cell cycle phase in which TRIM proteins are most involved is the mitosis. During mitosis TRIMs superfamily has been shown to exert important roles in the regulation of the main components of the mitotic spindle machinery, including kinetochores, centrosomes and midbodies that are important elements for ensuring chromosomes orientation and segregation to be performed correctly.

All the evidences collected and discussed in this review show that TRIM proteins may act as oncogenes or tumor suppressor genes, controlling cell proliferation and mitosis.

## Figures and Tables

**Table 1:** TRIMs’ roles in cancer. TRIM family members that control cell cycle progression have different role in controlling cancer development and progression.

TRIM	Type of Cancer	Role	Reference
TRIM2	ccRCC	tumor suppressor	[86]
TRIM3	CC	tumor suppressor	[87]
	HCC	tumor suppressor	[88]
TRIM8	GM	tumor suppressor	[43]
	RCC	tumor suppressor	[42]
	ATC	tumor suppressor	[34]
TRIM11	PC	oncogene	[70]
	OC	oncogene	[71]
	LC	oncogene	[72]
	HCC	oncogene	[73,74]
TRIM14	OS	oncogene	[41]
	GC	oncogene	[60]
	CRC	oncogene	[59]
	GBM	oncogene	[58]
TRIM15	GADC	tumor suppressor	[89]
	CRC	tumor suppressor	[90]
TRIM16	NB, LC, SC, BC	tumor suppressor	[63]
	HCC	tumor suppressor	[65]
	OC	tumor suppressor	[64]
TRIM23	GC	oncogene	[75]
TRIM24	CRC	oncogene	[76]
TRIM25	CRC	oncogene	[77]
	PC	oncogene	[78]
	GC	oncogene	[79]
TRIM26	HCC	tumor suppressor	[91]
TRIM27	CRC	oncogene	[54]
	IDC	oncogene	[55]
	OC	oncogene	[56,57]
TRIM28	GM	oncogene	[24]
	PC	oncogene	[44]
	HCC	oncogene	[46]
	BC	oncogene	[45]
TRIM29	CRC	oncogene	[25]
	NSCLC	oncogene	[50]
	LC	oncogene	[51]
	GC	oncogene	[52]
	SCC	tumor suppressor	[49]
TRIM31	HCC	oncogene	[80]
TRIM32	LC	oncogene	[99]
	GC	oncogene	[108]
	NB	tumor suppressor	[100]
TRIM36	PC	tumor suppressor	[92]
TRIM37	GM	oncogene	[81]
TRIM44	CRC	tumor suppressor	[101]
	PTC	oncogene	[103]
	PC	oncogene	[105]
	BC	oncogene	[104]
	NSCLC	oncogene	[106]
	TGCT	oncogene	[107]
	HEC	oncogene	[102]
TRIM45	GM	tumor suppressor	[93]
TRIM47	CRC	oncogene	[82]
	PC	oncogene	[83]
TRIM50	HCC	tumor suppressor	[94]
TRIM52	GM	tumor suppressor	[26]
	OC	oncogene	[48]
	HCC	oncogene	[27]
TRIM58	LADC	tumor suppressor	[96]
	CRC	tumor suppressor	[95]
TRIM59	NSCLC	oncogene	[29]
	CCA	oncogene	[66]
	BC	oncogene	[67]
	EOC	oncogene	[109,110]
	RCC	oncogene	[69]
	Bca	oncogene	[111]
	CRC	oncogene	[68]
	OS	oncogene	[112]
	PC	oncogene	[113]
	CC	oncogene	[28]
TRIM62	AML	tumor suppressor	[97]
TRIM65	UCB	oncogene	[84]
TRIM66	OS	oncogene	[30]
	NSCLC	oncogene	[61,62]
TRIM68	CRC	tumor suppressor	[31]
TRIM71	NSCLC	oncogene	[85]

ccRCC: clear cells renal carcinoma; CC: cervical cancer; HCC: hepatocellular carcinoma; GM: glioma; RCC: renal cell carcinoma; ATC: anaplastic thyroid carcinoma; PC: prostate cancer; OC: ovarian cancer; LC: lung cancer; OS: osteosarcoma; GC: gastric cancer; CRC: colorectal cancer; GBM: glioblastoma; GADC: gastric adenocarcinoma; NB: neuroblastoma; SC: skin cancer; BC: breast cancer; NSCLC: non-small cell lung carcinoma; IDC: intraductal carcinoma; SCC: squamous cell carcinoma; PTC: papillary thyroid cancer; TGCT: testicular germ cell tumor; HEC: human esophageal cancer; LADC: lung adenocarcinoma; CCA: cholangiocarcinoma; EOC: epithelyal ovarian cancer; Bca: bladder cancer; AML: acute myelod leukemia; UCB: bladder urothelial carcinoma).
